# Hospital Financial Health and Provision of Obstetric and Neonatal Intensive Care Unit Services

**DOI:** 10.1001/jamanetworkopen.2025.26418

**Published:** 2025-08-12

**Authors:** Elizabeth G. Salazar, Molly Passarella, Sara C. Handley, Jeannette Rogowski, Erika M. Edwards, Ciaran S. Phibbs, Scott A. Lorch

**Affiliations:** 1 Division of Neonatology, The Children’s Hospital of Philadelphia, Philadelphia, Pennsylvania; 2 Perelman School of Medicine at the University of Pennsylvania, Philadelphia; 3 Leonard Davis Institute of Health Economics, Philadelphia, Pennsylvania; 4 The Pennsylvania State University, University Park; 5 Vermont Oxford Network, Burlington; 6 Department of Pediatrics, The Robert Larner MD, College of Medicine, The University of Vermont, Burlington; 7 Department of Mathematics and Statistics, The University of Vermont, Burlington; 8 Veterans Affairs Palo Alto Health Care System, Palo Alto, California; 9 Stanford University School of Medicine, Stanford, California

## Abstract

This cohort study analyzes the association of hospital financial health with provision of obstetric and neonatal intensive care unit (NICU) services.

## Introduction

Access to high-quality US hospital–based perinatal care is limited, contributing to poor outcomes.^1^ Because perinatal care is often Medicaid-funded with high fixed costs for specialized units, its provision may create hospital financial strain.^2^ Strong hospital financial health, or a hospital’s long-term financial stability, is associated with improved adult care quality and outcomes, but its association with perinatal access and outcomes is unclear.^3^ We examined hospital provision of perinatal services stratified by hospital financial health, and the associated health policy of disproportionate share hospital (DSH) payments, which financially support hospitals serving high proportions of Medicaid patients.

## Methods

This retrospective cohort study followed the STROBE guideline and was determined exempt from review and the requirement of informed consent by the Children’s Hospital of Philadelphia institutional review board. We used 2010 to 2018 annual American Hospital Association (AHA) survey and Centers for Medicare & Medicaid Services Healthcare Cost Report Information System data (91% merge). The primary outcome was provision of perinatal service, defined by obstetric or neonatal beds: obstetric (OB) only, OB and neonatal intensive care unit (OB and NICU), NICU only, or neither service.^4,5^ Centers for Disease Control and Prevention (CDC) Wide-Ranging Online Data for Epidemiologic Research (WONDER) data provided 2018 state neonatal mortality rates. We obtained 2018 to 2021 birth parent mortality rates from the CDC National Vital Statistics System. The primary exposure was tertiles of winsorized hospital operating margin (OM).^3^ Secondary exposures were tertiles of the Yale Hospital Financial Score (YHFS), a validated composite of 10 financial measures.^6^ We examined associations of tertiles of financial health with perinatal services using ordinal logistic regression clustered by hospital with robust standard errors, unadjusted and adjusted for AHA hospital characteristics (bed number, ownership, year, teaching, rurality, critical access center, rural referral center, sole community hospital, and system affiliation). We analyzed the geographic distribution of hospitals with perinatal services by OM tertiles overlayed on state 2018 neonatal and 2018 to 2021 birth parent mortality rates. We also examined the correlation of percentage of births in hospitals receiving DSH payments with mortality. Additional information is available in the eMethods in Supplement 1. Analysis was conducted from May 2023 to June 2025 with significance defined as a 2-sided *P* < .05.

## Results

The study included 4931 hospitals, with 1164 providing OB and NICU care, 2026 providing OB care, 83 providing NICU care, and 2008 providing neither service. Low OM hospitals were more frequently government-owned, nonteaching, critical access, and located in southern and rural areas. These hospitals provided less OB and NICU care, had few births annually, had worse financial metrics, and were less likely to receive DSH payments (Table).

**Table.** Hospital Characteristics by Tertiles of Operating Margin

Characteristic	Hospitals, No. (%) (N = 4931)			
	Overall (n = 39 704)	Low (n = 12 989)	Moderate (n = 12 989)	High (n = 12 984)
General characteristics				
Total hospital beds, median (IQR)^a^	98 (30 to 229)	55 (25 to 153)	113 (36 to 247)	138 (50 to 275)
Total births/y				
≤500	23 727 (60)	9837 (76)	7534 (58)	5928 (46)
501-1000	5446 (14)	1185 (9)	1962 (15)	2234 (17)
1001-1999	5336 (13)	1055 (8)	1791 (14)	2378 (18)
≥2000	5195 (13)	912 (7)	1702 (13)	2444 (19)
Hospital ownership^a^				
Government	9029 (23)	4953 (38)	2582 (20)	1188 (9)
Not-for-profit	24 179 (61)	6560 (51)	8935 (69)	8274 (64)
Private	6496 (16)	1476 (11)	1472 (11)	3522 (27)
Teaching status^a^^,^^b^				
Major	2299 (6)	739 (6)	816 (6)	722 (6)
Minor	7799 (20)	1742 (13)	2613 (20)	3182 (25)
None	29 606 (75)	10 508 (81)	9560 (74)	9080 (70)
Sole community hospital^a^	3010 (8)	1210 (9)	929 (7)	867 (7)
Rural referral center^a^	1973 (5)	373 (3)	766 (6)	830 (6)
Critical access hospital^a^	11 528 (29)	5165 (40)	3920 (30)	2336 (18)
Region^a^				
Northeast	4919 (12)	1750 (14)	2136 (16)	1007 (8)
Midwest	12 115 (31)	3504 (27)	4497 (35)	4013 (30)
South	14 941 (38)	5437 (42)	4147 (32)	5227 (40)
West	7728 (20)	2297 (18)	2209 (17)	2737 (21)
System affiliation^a^	24 215 (61)	6022 (46)	7394 (57)	10 222 (79)
Rurality^a^^,^^c^				
Metropolitan	23 165 (58)	5979 (46)	7448 (57)	9269 (71)
Micropolitan	6604 (17)	2089 (16)	2375 (18)	2039 (16)
Noncore	9925 (25)	4916 (38)	3162 (24)	1675 (13)
Perinatal services^a^^,^^d^				
Obstetric and NICU	9957 (25)	1860 (14)	3265 (25)	4620 (36)
Obstetrics only	15 566 (39)	4530 (35)	5858 (45)	4899 (3)
NICU only	516 (1)	178 (1)	136 (1)	180 (1)
No obstetrics or NICU	13 665 (34)	6421 (50)	3730 (29)	3285 (25)
Financial outcomes^e^				
Operating margin, median (IQR), %^a^^,^^f^	−1.8 (−9.9 to −5.2)	−15.1 (−25.5 to −9.9)	−1.8 (−4.0 to 0.2)	8.7 (5.2 to 14.9)
Yale Hospital Financial Score, median (IQR)^a^^,^^g^	52.6 (33.3 to 67.2)	30.0 (21.5 to 46.0)	49.1 (38.1 to 58.7)	70.1 (61.6 to 77.5)
Total margin, median (IQR), %^a^^,^^h^	4.3 (−0.8 to 10.1)	−2.9 (−8.8 to 2.0)	3.0 (0.7 to 5.6)	12.3 (8.4 to 18.1)
Days’ cash on hand, median (IQR)^a^^,^^i^	18.0 (0.5 to 63.1)	19.4 (3.4 to 59.0)	26.3 (3.7 to 68.3)	8.6 (0.0 to 65.2)
Disproportionate share hospital payments^a^	23 490 (60)	6749 (52)	7760 (60)	8726 (67)
Current ratio, median (IQR)^a^^,^^j^	2.0 (1.2 to 3.3)	1.7 (0.9 to 3.0)	2.1 (1.3 to 3.2)	2.3 (1.4 to 3.8)
Net patient revenues, median (IQR)^a^	73 million (21 million 2.2 billion)	27 million (12 million to 1 billion)	88 million (3 million to 2.3 billion)	1.4 billion (54 million to 3 billion)

Abbreviation: NICU, Neonatal Intensive Care Unit.

^a^ Indicates *P* < .01 for all comparisons (low, medium, and high) in the category. A Kruskal-Wallis test was used for total beds and a Pearson χ^2^ test was used for total births, hospital ownership, teaching status, sole community hospital, rural referral region, critical access hospital, region, system affiliation, rurality, and perinatal services.

^b^ Major teaching is defined as a member of the Council of Teaching Hospitals and American Medical Colleges. Minor teaching is defined as having a Graduate Medical Education Council residency program without other teaching association memberships.

^c^ Rurality is defined using urban influence codes. Urban influence codes 1 or 2 are metropolitan; 3, 5, or 8 are micropolitan; and 4, 6, 7, 9, 10, 11 or 12 are noncore.

^d^ Obstetric level of care is defined by American Hospital Association data. NICU level of care is defined by a published validated list.

^e^ Centers for Medicare & Medicaid Services Healthcare Cost Report Information System data obtained from Wharton Research Data Services.

^f^ Operating margin is calculated by (net patient revenues less total operating expense) / net patient revenues × 100.

^g^ A composite measure of hospital financial health composed of 10 indicators.

^h^ Total margin is calculated by (net patient revenues less total operating expense + total other income) / net patient revenues × 100.

^i^ Days’ cash on hand is calculated by (cash on hand + temporary investments) / (total operating expense less depreciation) / 365.

^j^ Current ratio is calculated by total current assets / total current liabilities.

The highest OM tertile was associated with provision of OB and NICU, OB, and NICU care in unadjusted and adjusted models compared with the lowest tertile (OB and NICU: adjusted odds ratio [aOR], 2.21; 95% CI, 2.10-2.34; OB: aOR, 2.23; 95% CI, 2.09-2.38; NICU: aOR, 2.02; 95% CI, 1.86-2.18). Similar associations were found with YHFS (OB and NICU: aOR, 1.72; 95% CI, 1.64-1.82; OB: aOR, 1.60; 95 CI, 1.51-1.70; NICU: aOR, 2.02; 95% CI, 1.88-2.18).

We examined the geographic distribution of perinatal-serving hospitals by OM tertile overlayed with state neonatal and birth parent mortality rates (Figure). States with a higher percentage of births at DSH-receiving hospitals had a moderate negative correlation with birth parent (Spearman ρ = −0.60; *P* < .001) but not neonatal mortality rates (Spearman ρ =  −0.01; *P* = .93).

**Figure.**
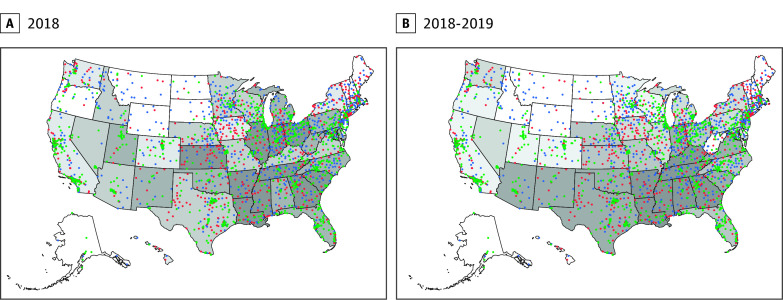
Perinatal-Serving Hospitals by Financial Health Over 2018 Neonatal or 2018-2019 Birthing Parent Mortality Rates Shading of states indicates quintiles of 2018 neonatal mortality rate (A; Centers for Disease Control and Prevention [CDC] Wide-Ranging Online Data for Epidemiologic Research ) or quartiles of 2018-2021 birthing parent mortality rate (B; CDC National Vital Statistics System) with higher mortality rates indicated in darker shading and lower rates indicated in lighter shading. Dot color indicates the tertile of operating margin for the hospital among all obstetric and/or Neonatal Intensive Care Unit serving hospitals nationally; green indicates the highest tertile; blue, the middle tertile; and orange, the lowest tertile.

## Discussion

In this cohort study, worse hospital financial health was associated with decreased likelihood of providing perinatal services. These hospitals were more frequently located in southern and rural areas. There was a moderate correlation with higher percentage of births in DSH-receiving hospitals and reduced maternal mortality. Limitations include residual confounding by unstudied covariates associated with poorer financial health, such as other hospital service lines, and the need for additional patient-level birth variables to evaluate whether decreased perinatal services aligned with changing community needs. Future work should examine how the lower profitability of perinatal services contributes to overall hospital financial health, whether this association influences perinatal access and outcomes, and the role of DSH policies in mediating these associations.

## Notes

Supplement 1.
eMethods.

Supplement 2.
Data Sharing Statement
