# Supplementary material for: Hospital Financial Health and Provision of Obstetric and Neonatal Intensive Care Unit Services
Source: JAMA Netw Open. 2025 Aug 12;8(8):e2526418. doi: 10.1001/jamanetworkopen.2025.26418 (PMC12344535; doi:10.1001/jamanetworkopen.2025.26418)
Supplement: Supplement 1. — eMethods. [file jamanetwopen-e2526418-s001.pdf]

Supplemental Online Content

Salazar EG, Passarella M, Handley SC, et al. Hospital financial health and provision of obstetric and neonatal intensive care unit services. *JAMA Netw. Open*. 2025;8(8):e2526418. doi:10.1001/jamanetworkopen.2025.26418

eMethods.

This supplemental material has been provided by the authors to give readers additional information about their work.

© 2025 Salazar EG et al. *JAMA Network Open*.

eMethods

Analyses were performed using Stata, version 17 (StataCorp). This study followed the STROBE reporting guidelines and was determined exempt by the Children's Hospital of Philadelphia IRB. We created a chloropeth map with a base of quintiles of 2018 infant mortality rate or 2018-2021 maternal mortality rate overlayed with hospitals colored by tertiles of hospital financial health, measured in operating margin using R version X (R Project for Statistical Computing).

Financial Measures

All financial measures were obtained from Centers for Medicare and Medicaid Services (CMS) Healthcare Cost Report Information System data, available through Wharton Research Data Services. Definitions of specific measures are seen below:

-Operating Margin: (Net patient revenues less total operating expense) / net patient revenues * 100

-Yale Hospital Financial Score: Standardized score on hospital financial health calculated using methods described by Zinoviev et al., 2021, including validation against bond ratings. Components include profit margin, current debt service coverage, days cash on hand, return on assets, total debt / capitalization, return on equity, net patient revenue, interest coverage, long term debt / capitalization %, salaries and benefits

-Disproportionate Share Hospital (DSH) Payments: DSH payments are federal funds allocated to states to offset costs incurred by hospitals serving a high number of Medicaid and uninsured patients. DSH payments help these hospitals provide care to vulnerable populations while mitigating the financial burden of treating those who are uninsured or underinsured. There is state level variation in criteria for receiving DSH payments.

-Total Margin: (Net patient revenues less total operating expense + total other income) / (net patient revenue + total other income) * 100.

-Days Cash on Hand: (Cash on hand + temporary investments) / ((total operating expense less depreciation) / 365).

-Current Ratio: Total current assets / total current liabilities.

-Net Patient Revenues: Total patient revenue less allowances and discounts on patient accounts.

Neonatal Mortality Rates

Neonatal deaths were defined as deaths from birth to 27 days of life. This definition was chosen as it would most plausibly be related to changes in hospitals care. This data was obtained from the Centers for Disease Control and Prevention (CDC) Wide-ranging Online Data for Epidemiologic Research (WONDER) database.

Maternal Mortality Rates

Maternal deaths were defined as "deaths of women while pregnant or within 42 days of termination of pregnancy, irrespective of the duration and the site of the pregnancy, from any cause related to or aggravated by the pregnancy or its management, but not from accidental or incidental causes." This definition was chosen as it would most plausibly be associated with changes in hospital aspects of care. This data was obtained from the Kaiser Family Foundation, with the original source being the Center for Disease Control and Prevention, National Center for Health Statistics, National Vital Statistics System: https://www.cdc.gov/nchs/maternal-mortality/MMR-2018-2022-State-Data.pdf.

© 2025 Salazar EG et al. *JAMA Network Open*.

Consort Diagram of Study Cohort Creation

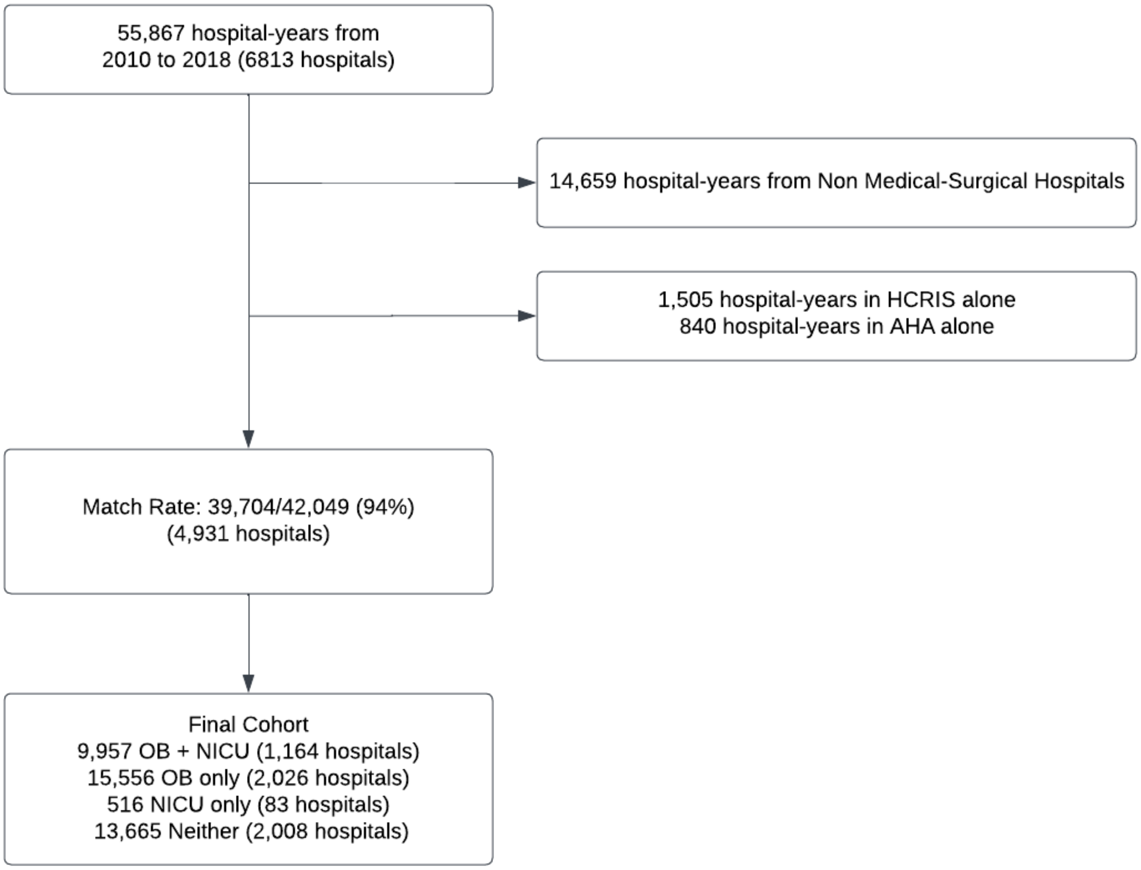

© 2025 Salazar EG et al. *JAMA Network Open*.

Study Variable or Outcome	Administrative Data Source
Hospital Type	AHA serv variable Hospitals with the following codes were included:<br>10 General medical and surgical<br>44 Obstetrics and gynecology<br>50 Children's general medical and surgical
Total hospital beds	American Hospital Association
Births per year	American Hospital Association
Hospital Ownership	American Hospital Association
Teaching Status	American Hospital Association
Community Hospital Designation	American Hospital Association
Rural Referral Center	American Hospital Association
Critical Access Hospital	American Hospital Association
Census Region	American Hospital Association
Urban Influence Code	American Hospital Association
Bassinet Number	American Hospital Association
NICU Beds	American Hospital Association
Intermediate Care Beds	American Hospital Association
Part of a Larger System	Manually identified using American Hospital Association data
Obstetric Beds	American Hospital Association
Obstetric Service Status	Manually identified using methods from reference (31)
NICU Status	Manually identified using reference (32)
Obstetric level of Care	American Hospital Association
NICU level of care	Manually identified using reference (32)
Children's Hospital	Manually identified using reference (32)
Operating Margin	Cleaned HCRIS data from WRDS
Yale Hospital Financial Score	Calculated using methods from reference (18) using cleaned HCRIS data from WRDS
Total Margin	Cleaned HCRIS data from WRDS
Days Cash on Hand	Cleaned HCRIS data from WRDS
DSH Payments	Cleaned HCRIS data from WRDS
Current Ratio	Cleaned HCRIS data from WRDS
Net Patient Revenues	Cleaned HCRIS data from WRDS
State 2018 Neonatal Death Rates	2018 CDC Wide-ranging ONline Data for Epidemiologic Research (WONDER) Data
State 2018-2021 Maternal Mortality Rates	This data was obtained from the Kaiser Family Foundation, with the original source being the Center for Disease Control and Prevention, National Center for Health Statistics, National Vital Statistics System: https://www.cdc.gov/nchs/maternal-mortality/MMR-2018-2022-State-Data.pdf.

© 2025 Salazar EG et al. *JAMA Network Open*.
