# Supplementary material for: Hospital Financial Health and Provision of Obstetric and Neonatal Intensive Care Unit Services
Source: JAMA Netw Open. 2025 Aug 12;8(8):e2526418. doi: 10.1001/jamanetworkopen.2025.26418 (PMC12344535; doi:10.1001/jamanetworkopen.2025.26418)
Supplement: Supplement 2. — Data Sharing Statement [file jamanetwopen-e2526418-s002.pdf]

Data Sharing Statement

Salazar. Hospital Financial Health and Provision of Obstetric and Neonatal Intensive Care Unit Services in the US. *JAMA Netw Open*. Published August 12, 2025. doi:10.1001/jamanetworkopen.2025.26418

Data
Data available: No

Additional Information
Explanation for why data not available: Portions of data are proprietary with Wharton Research Data Services and may be made available if the individual has a data use agreement with this entity.
